# Miscellaneous Paragraphs

**Published:** 1893-08

**Authors:** 


### LIBERAL USE OF BUTTER.

No dietetic reform would be more conducive to improve health among children, and especially to the prevention of tuberculosis, than an increase in the consumption of butter, says an exchange. Our children are trained to take butter with great restraint, and are told that it is greedy and extravagant to eat much of it. It is regarded as a luxury, and as giving a relish to bread rather than in itself a most important article of food. Even to private families of the wealthier classes these rules prevail at table, and at schools, and at public boarding establishments they receive strong reinforcements from economical motives. Minute allowances of butter are served out to those who would gladly consume five times the quantity. Where the house income makes this a matter of necessity there is little more to be said than that it is often a costly economy. Enfeebled health may easily entail a far heavier expense than a more liberal breakfast would have done.

Cod liver oil costs more than butter, and it is, besides, often not resorted to until too late. Instead of restricting a child's consumption of butter, encourage it. Let the limit be the power of digestion and the tendency to biliousness. Most children may be allowed to follow their own inclinations and will not take more than is good for them. The butter should be of the best and taken cold. Bread, dry toast, biscuits, potatoes and rice are good vehicles. Children well supplied with butter feel the cold less than others and resist the influenza better. They do not "catch cold" so easily. In speaking of children, I by no means intend to exclude other ages, especially young adults. Grown-up persons, however, take other animal fats more freely than most children do, and are, besides, allowed much freer selection as to both quality and quantity. It is not so necessary to raise any clamor for reform on their account.

### BREAD AND DYSPEPSIA.

The conclusion that wheat bread is unfit for dyspeptics, sometimes jumped at because ill effects are noticed to follow its use, is erroneous. On the contrary it has been pointed out by Bouchard and others, that farinaceous food is peculiarly adapted to some dyspeptic patients. It is the microbes in the starch, which are capable of producing irritating acids, that cause the trouble. To avoid this, Bouchard recommends that only the crust or toasted crumb of the bread be used by dyspeptics, particularly those whose stomachs are dilated. The reason of this is explained by the fact that baking, temporarily, though not permanently, arrests the fermentation of dough. When it is again heated by the warmth of the stomach the fermentation is renewed. In cases where the bread is toasted brown through the fermentation is stopped permanently.

### THEORY OF HUMAN ACTION.

Every human action has three aspects: its moral aspects, or that of its right and wrong; its æsthetic aspect, or that of its beauty; its sympathetic aspect, or that of its loveableness. The first addresses itself to our reason and conscience; the second to our imagination; the third to our human fellow-feeling. According to the first, we approve or disapprove; according to the second, we admire or despise; according to the third, we love, pity or dislike. The morality of an action depends upon its foreseeable consequences; its beauty and its loveableness, or the reverse, depend upon the qualities which it is evidence of. Thus, a lie is wrong. because its effect is to mislead, and because it tends to destroy the confidence of man in man; it is also mean, because it is cowardly; because it proceeds from not daring to face the consequences of telling the truth; or, at best, is evidence of that want of power to compass our ends by straightforward means, which is conceived as properly belonging to every person not deficient in energy or in understanding. The action of Brutus in sentencing his sons was right, because it was executing a law essential to the freedom of his country against persons of whose guilt there was no doubt; it was admirable, because it evinced a rare degree of patriotism, courage, and self-control; but there was nothing loveable in it; it affords no presumption in regard to loveable qualities, unless a presumption of their deficiency. If one of the sons had engaged in the conspiracy from affection for the other, his action would have been loveable, though neither moral nor admirable. It is not possible for any sophistry to confound these three modes of viewing an action, but it is very possible to adhere to one of them exclusively, and lose sight of the rest. Sentimentality consists in setting the last two of the three above the first; the error of moralists in general, is to sink the two latter entirely.

---

### OVER-EATING.

Not every one over-eats; but some do; to them this brief paragraph is addressed. An excess of food causes an embarrassment to the digestive organs; decomposition and flatulence set in, bacteria multiply and add to the trouble. Putrid and more or less toxic gases and ptomaines, especially if much flesh is eaten, are generated, and a bilious condition supervenes. That sleeplessness should attend such a state of affairs is not surprising. The remedy for this is to reduce the daily rations to the bodily requirements. The necessity of eating slowly and deliberately is apparent, as rapid eaters are more likely to over-eat. Poor food may engender sleeplessness by inducing anaemia of brain and other organs. It cannot be too much insisted upon that the daily fare contain an adequate mixture of albumen, fats and carbohydrates. Indigestible food produces the same evils as excessive amounts of food. Under this head may be ranked improperly cooked food, unripe fruit, pastries, hot bread, fried pork, confectionery. Foods which alone are digestible may become indigestible if too many kinds are eaten to a meal.

The peculiarities of the individual must be respected, and indigestible articles avoided. Much depends upon the muscular work done. Thus haymakers on the salt marshes need food of hard digestion, so as to yield force slowly during many hours; eat with impunity baked beans, boiled beef, cabbage, etc. These people sleep well in spite of their abominable fare. Such a diet would upset a brainworker or person of sedentary habits. A healthy digestion presupposes a healthy state of the stomach, intestines and accessory organs, and any derangements of these viscera must be corrected before normal sleep can be enjoyed.

### SEA-BATHING.

Sea-bathing, on account of its stimulating and penetrating power, may be placed at the head of those means that regard the care of the skin, and which certainly supplies one of the first wants of the present generation, by opening the pores, and thereby reinvigorating the whole nervous system. This bathing is attended with two important advantages. The first is, that besides its great healing power in cases of disease, it may be employed by those who are perfectly well, as the means most agreeable to nature for strengthening and preserving health. In this respect it may be compared to bodily exercise, which can remove diseases otherwise incurable, and which may be used also by those who are sound in order to preserve themselves in that state. The other advantage is, the noble, grand, and indescribable prospect of the sea connected with it, and which, on those not acquainted with it, has an effect capable of bracing up the nervous system and producing a beneficial exaltation of the whole frame. I am fully convinced that the physical effects of sea-bathing must be greatly increased by this impression on the mind, and that a hypochondriac or nervous person may be half-cured by residing on the sea-coast, and enjoying a view of the grand scenes of nature which will there present themselves.

### WRINKLES.

No evidence of advancing years is so unwelcome as wrinkles. Wrinkles are but the expression of the inner life, thought, and feeling as the years go by. The best remedies for wrinkles are altogether preventive. A cheerful spirit, contented mind, plenty of sleep, exercise in the open air, and a healthful diet will insure against the appearance of wrinkles almost entirely. When once developed, these measures stand among the best cures known. We may, however, aid in the removal of these unwelcome marks of advancing years by sponging the the face with hot water and daily taking a face massage with some oil that is readily absorbed, as lanoline or malvena salve.

These lines are traced as the expression of the face gives them existence, and are encouraged by the absorption of the fat which underlies the skin; when this is developed by massage, the removal of the wrinkles is hastened, but the principal thing to be attained is to prevent their existence by a change of thought, condition of mind and heart.

### WASTE OF FORCE.

A source of dyspepsia is emotional waste of nervous force. The nerve force is to the physical system what steam is to the machine. In the normal condition of things, it is renewed as fast as it is used. But nature makes no provision for the immense amount expended by excessive care, by fuss and worry, by hurry and drive, by explosions of passion and by the undue excitements of pleasure. All these are like a great leakage of steam. The stomach is the first and largest sharer in the loss.

### HICCOUGH.

Hiccough in most cases is a very trifling trouble, which generally yields readily to any one of many simple expedients, but in rare instances it proves intractable. A little sugar water quickly overcomes it in babies, while a few swallows of water usually do the same for adults.

Persistent hiccough which stoutly resists treatment, occurs only among the latter, and generally late in the course of fatal chronic diseases in which the stomach or liver is involved. In such cases subcutaneous injections of medicines and galvanism are among the remedies generally employed. A very simple remedy, worth recording, has recently proved effectual after all the ordinary measures were resorted to without avail. It was merely a teaspoonful of pulverized sugar wet with same quantity of vinegar. This was taken at one dose, and immediately stopped the hiccough, which did not return for six hours, and then ceased after a second dose of the same remedy.

### INFECTION FROM SOIL.

Much of the ills, whose origin is seemingly unknown, incident to living in rural communities, may be traced to their very doorsteps for their starting place. In how many places is the soil without the kitchen soaked with slops from the cooking, dish washing, etc? Three times a day for weeks, months and years, may be, the ground has been made to serve for a drain pipe. Filtration after a while becomes impossible owing to the saturated condition of the ground, which is now in a fine condition for breeding bacteria. The slops flow on—the bacteria are caught up in the flow and finally the dirty, germ-laden water, finds its way into a well, occasioning deadly diseases.

### LIME JUICE BETTER THAN VINEGAR.

Lime juice is very similar to lemon juice in its nature, and is sold in the market by the bottle. It is generally acknowledged to be an antidote to scurvy, and by English law it is rendered compulsory for every ship to take on board lime or lemon juice. For the Navy the Admiralty use lime juice only. The constant use of lime or lemon juice of good quality greatly discourage a number of complaints—such as tumor, cancer, dyspepsia, bilious disorders, etc., which the present luxurious state of living on liberal flesh, alcoholic diet, without its corrective aid, greatly fosters. This vegetable acid should be placed on the dinner table instead of the vinegar bottle, and as regularly as salt, while as an ingredient for sauce, for almost any kind of food, it has no equal. As a salad dressing to mix with oil, it is more wholesome than vinegar.

### HOW TO FUMIGATE A ROOM.

The proper way to fumigate a room is to close the doors, windows, fireplace, etc., pasting strips of paper over all the cracks. Fumigation by burning sulphur is most easily accomplished. Two pounds of sulphur should be allowed for every room from ten to twelve feet square. It is better to divide it up and put it in several pans, rather than burn the entire quantity of sulphur used in one pan. To avoid the danger of fire, these pans should be set on bricks, or in other and larger pans filled with water or with sand. After pouring a little alcohol on the sulphur and properly placing the pans about the room, the furthest from the door of exit should be lighted first, the others in order. The operator will need to move quickly, for no one can breathe sulphurous flames with safety. After closing the door, the cracks around it should be pasted up as was done within the room. Six hours at least is generally necessary to fumigate a room properly; at the end of that time it may be entered and the windows opened; and they should be left open as long as is convenient, even for a week if possible. After fumigation, a thorough process of cleansing should be instituted. At least the walls and ceiling should be rubbed dry; much the better way is to whitewash and re-paper. The floor and the woodwork and the furniture should be scrubbed with a solution of carbolic acid or some other disinfectant.

### PERSPIRATION.

If you perspire excessively, avoid warm baths, and if unable to take absolute cold ones, sponge the body with slightly tepid water, to which has been added diluted sulphuric acid in a proportion of two drams to a pint of water. The affected parts should then be powdered generously with powdered starch, which could be scented with orris root.

The foot-sore wanderer through the World's Fair buildings will find solace in a foot bath of hot alum water every night before going to bed. With the best of care the feet are apt to be tender and troublesome in the warm months, and this will be found most efficacious.

